# Analysis of filtration behavior using integrated column chromatography followed by virus filtration

**DOI:** 10.1002/bit.27840

**Published:** 2021-06-08

**Authors:** Hironobu Shirataki, Yoshiro Yokoyama, Hiroki Taniguchi, Miku Azeyanagi

**Affiliations:** ^1^ Bioprocess Division, Scientific Affairs Group Asahi Kasei Medical Co., Ltd. Tokyo Japan; ^2^ Bioprocess Division, Department of Technology Asahi Kasei Medical Co., Ltd. Nobeoka‐shi Miyazaki‐ken Japan; ^3^ Bioprocess Division, Department of New Products Development Asahi Kasei Medical Co., Ltd. Fuji‐shi Shizuoka Japan

**Keywords:** chromatography, integrated process, virus filtration

## Abstract

We evaluated filtration behavior and virus removal capability for a monoclonal antibodies (mAb) and plasma IgG under constant flow rate directly following flow‐through column chromatography in an integrated process. mAb solution with quantified host cell protein (HCP) content processed in flow‐through mode on in‐series mixed‐mode AEX and modified CEX columns connected to the Planova BioEX filter (pool‐less) achieved HCP logarithmic reduction value (LRV) of 2.3 and 93.9% protein recovery, demonstrating comparable or higher HCP LRV with high protein recovery compared to previous reports. For 5–15 mg/ml plasma IgG run to 100 L/m^2^, similar filtration behavior was achieved for flux of 10–100 LMH, and lower flux runs remained well below the maximum operating pressure, suggesting that higher throughput in continuous processing is achievable. Comparison of fit of plasma IgG and mAb filtration behavior to four blocking models showed little differences but slightly better fit to the cake filtration model. Viral clearance of the filtration step tested by in‐line spiking X‐MuLV or MVM into purified plasma IgG following the chromatography step showed robust removal at low flux. Integrating the Planova BioEX filter into continuous processes with column chromatography can achieve efficient downstream processing with reduced footprint and process time.

## Graphical abstract

The authors designed and tested a total flow‐through integrated process in which selected AEX and CEX columns were combined with virus filtration to achieve good HCP reduction and protein recovery. Transmembrane pressure was stable for the virus filter, and in‐line virus spiking tests demonstrated robust viral clearance in the integrated process. Robust viral clearance was observed even for very low flux filtrations with process pause, and blocking models were used to characterize virus filtration properties.

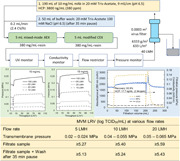

## 1 INTRODUCTION

The growth of the biologics market since 2000 has been driven by the increasing production of monoclonal antibodies (mAb), which is in turn driving the demand for the development of more efficient upstream processes (USP) and downstream processes (DSP). Significant progress has been made in USP, resulting in remarkable improvements in space and time use in the past 10 years (Halan & Minas, 2020; Pollock et al., 2013; Rader & Langer, 2015). However, the lack of comparable advances in DSP is reported to have caused a bottleneck in production efficiency (Gronemeyer et al., 2014; Konstantinov & Cooney, 2015). One approach to address this bottleneck is to transition the production of biologics from batch processes with intermediate pool tanks with process pauses between each step to continuous processes with more efficient and steady production. For example, perfusion cell culture that allows continuous clarification of cell culture (Pollock et al., 2013; Schmidt, 2017; Schmidt & Wieschalka, 2017) can be paired with simulated moving‐bed (SMB) technology that allows continuous chromatography (Warikoo et al., 2012). Likewise, low pH virus inactivation is usually conducted in batch mode, but progress has been made to convert this step to a continuous process (Klutz et al., 2016). Despite these developments in biologics production, there are few commercial biologics produced by fully continuous processes due to the extreme difficulty in stabilizing and integrating each step of the process. In addition to process control issues, methodology for confirming the viral clearance for constant flow rate integrated processes should also be considered. Alternatively, hybrid systems that incorporate batch and continuous processes in production steps from cell culture to protein purification have been identified as a practical option and such integrated processes are being evaluated.

While batch processing at constant pressure may be preferred due to ease of control of the process, the final virus filtration can be integrated with column chromatography and operated under a constant flow rate. The development of the technology for implementing virus filtration in continuous processes is ongoing. One challenge is balancing the throughput capacity of column chromatography (column volume) and virus filter (effective surface area), as well as the flow rate across the system. The robustness of the virus filtration including any process pauses that may occur when switching feed stocks must be verified. Additionally, since column chromatography is generally conducted at a constant flow rate, it is necessary to conduct long‐duration constant flow rate virus filtrations. Another challenge to overcome is characterizing filtration stability. Virus filters are generally used in constant pressure mode, so there are concerns about pressure stability in long‐duration constant flow rate filtrations (Halan & Minas, 2020). Specifically, increased filtration pressure due to pore clogging and virus breakthrough due to low filtration pressures arising from the low flow rates are concerns. In a continuous process with a constant filtration pressure of 0.1–0.5 bar for 24–96 h and a process pause for a mAb with relatively low concentration (0.3 g/L), stable filtration and virus removal were achieved (Kleindienst et al., 2019). Long‐duration virus filtration of PP7‐spiked IgG for 4 days with Planova 20N and BioEX filters showed robust PP7 removal rates for both filter types even when challenged with a simultaneous spike in protein concentration, conductivity, and PP7 concentration to simulate feed composition changes that may occur during a preceding chromatography step (Lute et al., 2020). These studies suggest that although long‐duration processes under typical process conditions may be difficult to achieve, there are ways to implement virus filtration into continuous processes.

While integrated processes running at a constant flow rate are capable of reducing process footprint and processing time, it is necessary to evaluate the stability of virus filtration before adoption in production processes. In this study, we focus on the evaluation of integrated column chromatography and virus filtration steps. Host cell protein (HCP) removal and mAb recovery were evaluated for the integrated process with AEX and CEX chromatography directly connected to virus filtration. Additionally, a viral clearance test was conducted at a constant flow rate by in‐line spiking MVM or X‐MuLV to plasma IgG in an integrated process with AEX or CEX chromatography in series with virus filtration, in which the virus was spiked after the chromatography step and before the virus filtration step. The studies included a process pause followed by a buffer wash. Virus filtration of MVM‐spiked plasma IgG was conducted at low constant flow rates over long durations with a process pause to evaluate the effects of low flow rates and long durations. Finally, filtration behavior at a constant flow rate was theoretically investigated using blocking models for plasma IgG and mAb filtration runs.

## 2 MATERIALS AND METHODS

### 2.1 Monoclonal antibody (mAb)

The mAb used in this study was provided by Manufacturing Technology Association of Biologics (MAB), Japan. The mAb (pI 8.5) was grown in CHO cell culture by fed‐batch method (mAb concentration, 3.5 mg/ml; HCP concentration, about 80,000 ng/ml), separated from cells by a depth filter (Millistak + D0HC, MilliporeSigma) and applied to a bind‐elute affinity chromatography column with Protein A resin (MabSelect SuRe, GE Healthcare). The mAb solution purified by Protein A resin (mAb concentration, 30 mg/ml; HCP concentration, about 9000 ng/ml) was diluted with buffer (20 mM Tris‐Acetate, 100 mM NaCl; pH 5, 6, 7, or 8) to produce 10 mg/ml mAb solution with HCP concentration of about 1500 or 3000 ng/ml for use in preliminary experiments for chromatography column selection. A 10 mg/ml mAb solution with the same buffer at pH 6.5 with HCP concentration of 3800 ng/ml was prepared for processing in an integrated process with column chromatography and filtration with a virus filter. HCP in the mAb solution was analyzed using CHO Host Cell Protein ELISA 3 G Kit (Cygnus Technologies). Protein recovery was evaluated by comparing the absorbance at 280 nm before and after column chromatography using a SPECTRA max plus 384 (Molecular Devices, LLC) under the assumption that HCP, which has a much smaller concentration, does not affect UV absorbance.

### 2.2 Plasma IgG

Commercial venoglobulin IH5 (50 mg/ml) provided by Japan Blood Products Organization (JBPO) was used without further purification and was diluted to 5, 10, or 15 mg/ml in 20 mM sodium acetate, 100 mM NaCl, pH 5.0 for use in filtration experiments. For runs with plasma IgG solution spiked with virus, 5 mg/ml plasma IgG in 20 mM Tris‐Acetate, 100 mM NaCl, pH 6.5 with 1% virus spike (MVM or X‐MuLV) was used. TCID_50_ assay was conducted to measure the virus titer in the load solution and processed solution.

### 2.3 Chromatography resins

Preliminary experiments were conducted to evaluate HCP removal and protein recovery for mAb solutions under flow‐through mode for each type of AEX and CEX resin. The resins that performed best were used in the integrated column chromatography and virus filtration study. Resin type and its ligand, ion exchange capacity, and product name used in the experiments are as follows. For AEX resins, normal AEX (strong AEX; ion exchange capacity, 0.13–0.22 mmol/ml; Cellufine MAX Q‐h) and mixed‐mode AEX (weak AEX of primary amine in combination with weak HIC of butyl group; ion exchange capacity, 0.09–0.22 mmol/ml; Cellufine MAX IB) were evaluated. For CEX resins, normal CEX (strong CEX; ion exchange capacity, 0.09–0.21 mmol/ml; Cellufine MAX S‐r), grafted CEX (grafted chains with strong CEX ligands fixed on porous beads; ion exchange capacity, 0.09–0.15 mmol/ml; Cellufine MAX GS) and modified CEX (heparin mimetic resin with dextran sulfate ligands; ion exchange capacity, 0.036–0.07 mmol/ml; Cellufine DexS‐HbP) were used. All resins are supplied by JNC Corporation.

### 2.4 Virus filter

For filtration of both plasma IgG and mAb solutions, 0.0003 m^2^ Planova BioEX filters (Asahi Kasei Medical) were used.

### 2.5 Integrated system control with AKTA

The AKTA pure 25 or the AKTA avant 25 (GE Healthcare) was used to control the filtration and/or chromatography column processes. AKTA pure 25 was used for all studies except the plasma IgG filtration. The chromatography column(s) were connected to the column valve and the virus filter was connected to the outlet valve. For the integrated mAb process, the filter was positioned after the flow restrictor. AKTA avant 25 was used for plasma IgG filtration by connecting the filter to the column valve. The flow restrictor was removed to ensure that the pressure monitor displays filtration pressure, and pressure was recorded using the PreC pressure monitor on the AKTA avant 25.

### 2.6 Processing mab solution in an integrated process

In a preliminary study, AEX (normal AEX and mixed‐mode AEX) and CEX (normal CEX, grafted CEX and modified CEX) resins were evaluated individually for protein recovery and HCP removal using 10 ml of 10 mg/ml mAb in 20 mM Tris‐Acetate, 100 mM NaCl at pH 5, 6, 7, or 8 with HCP concentration of about 3000 ng/ml (for AEX resins) or 1500 ng/ml (for CEX resins) packed in 0.5 ml Tricorn 5/20 columns (5 mm ID; GE Healthcare) with a flow rate of 0.25 ml/min (0.5 CV/min). The load on each resin was 200 mg mAb/ml‐resin and 0.06 mg HCP/ml‐resin for AEX or 0.03 mg HCP/ml‐resin for CEX. The HCP logarithmic reduction value (HCP LRV) and protein recovery were determined by analyzing the flow‐through fraction (10 ml load and 2 ml wash, combined). Based on the protein recovery and HCP reduction results, mixed‐mode AEX and modified CEX were selected for use in the integrated process study conducted with buffer at pH 6.5.

**Figure 1:**
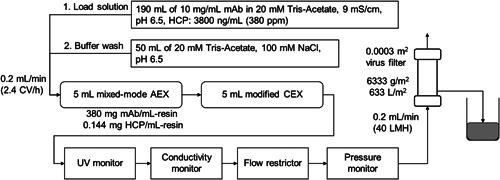
Setup for integrated process consisting of mixed‐mode AEX, modified CEX and a virus filter connected in series for processing of mAb solution (10 mg/ml mAb in 20 mM Tris‐acetate, 9 mS/cm, pH 6.5). AKTA pure 25 was used for this setup

For the integrated process setup, mixed‐mode AEX and modified CEX prepacked in Mini Column 5LM (14.6 mm ID, 30 mm L; CV of 5 ml; JNC Corporation) were run in‐series in a pool‐less integrated process with a 0.0003 m^2^ Planova BioEX filter on an AKTA pure 25 as shown in Figure 1. Mixed‐mode AEX and modified CEX were selected as the best combination for the integrated process with pH 6.5, and this selection process is further described in Section 3. Washing and equilibration with equilibration buffer (20 mM Tris‐Acetate, 100 mM NaCl, pH 6.5) were conducted on the chromatography columns and filter independently. The system was filled with equilibration buffer, and 190 ml of 10 mg/ml mAb solution with 3800 ng/ml HCP (380 ng/mg‐mAb) at 9 mS/cm was loaded on the system (380 mg mAb/ml‐resin and 0.144 mg HCP/ml‐resin) and at 633 L/m^2^ (6333 g/m^2^) to the filter, followed by 50 ml of equilibration buffer wash. The system ran with a flow rate of 0.2 ml/min (2.4 CV/h) and 40 LMH to the filter. HCP LRV and protein recovery were determined for the mAb flow‐through fraction and for the flow‐through fraction with the wash flush.

**Table 1:** Equations expressing the relationship between filtration volume and pressure for constant flow rate filtrations for the four blocking models

Cake filtration	Intermediate blocking	Standard blocking	Complete blocking
$p=p_{0}(1+\mathrm{kV})$ (1)	$p=p_{0}\exp(\mathrm{kV})$ (3)	$p=\frac{p_{0}}{{(1-\mathrm{kV}/2)}^{2}}$ (5)	$p=\frac{p_{0}}{\left(1-\mathrm{kV}\right)}$ (7)
$\frac{p}{p_{0}}-1=\mathrm{kV}$ (2)	$\ln\left(\frac{p}{p_{0}}\right)=\mathrm{kV}$ (4)	$2\left\{1-{\left(\frac{p_{0}}{p}\right)}^{1/2}\right\}=\mathrm{kV}$ (6)	$1-\frac{p_{0}}{p}=\mathrm{kV}$ (8)

Note: *p*_0_: the initial transmembrane pressure, *p*: the transmembrane pressure at the filtration volume, *V, k*: the plugging constant.

### 2.7 Filtration of plasma IgG solution with a virus filter

Plasma IgG solution filtration experiments were conducted to determine the pressure dependence of flux for the virus filter. After washing and equilibration with 20 mM sodium acetate, 100 mM NaCl, pH 5.0 buffer, plasma IgG solution (5, 10, or 15 mg/ml) in 20 mM sodium acetate, 100 mM NaCl buffer at pH 5.0 was filtered at constant flow rates corresponding to 10, 20, 50, or 100 LMH on a 0.0003 m^2^ Planova BioEX filter to a target throughput of 100 L/m^2^. Filtration was conducted using AKTA avant 25 (GE Healthcare). After prefiltering the sample solution with a 0.2 µm microfilter (Minisart RC 25 mm, Sartorius), the solution was filled into Superloop 150 ml (GE Healthcare), and the solution was loaded to the virus filter using the system pump.

### 2.8 Virus removal capabilities of column chromatography

Virus removal capabilities of mixed‐mode AEX, grafted CEX, and modified CEX packed in Tricorn 5/20 column (CV, 0.5 ml; 5 mm ID; GE Healthcare) were evaluated in flow‐through mode individually without a virus filtration step using AKTA pure 25. The viral clearance study was conducted by ViruSure (Vienna, Austria). The total load solution was 30 ml of 5 mg/ml plasma IgG (300 mg plasma IgG/ml‐resin) in 20 mM Tris‐Acetate, 100 mM NaCl, pH 6.5 with 5% virus spike and was passed through 0.45 μm PES prefilter before use and was loaded with flow rate of 0.25 ml/min (0.5 CV/min) by sample pump. Virus assay was conducted on both the first 15 ml flow‐through fraction (load capacity of 150 mg/ml‐resin) and the total 30 ml flow‐through fraction (load capacity of 300 mg/ml‐resin). Log titers of load solution were 7.7 and 6.6 TCID_50_/ml for MVM and X‐MuLV, respectively.

### 2.9 Viral clearance for integrated system with plasma IgG

**Figure 2:**
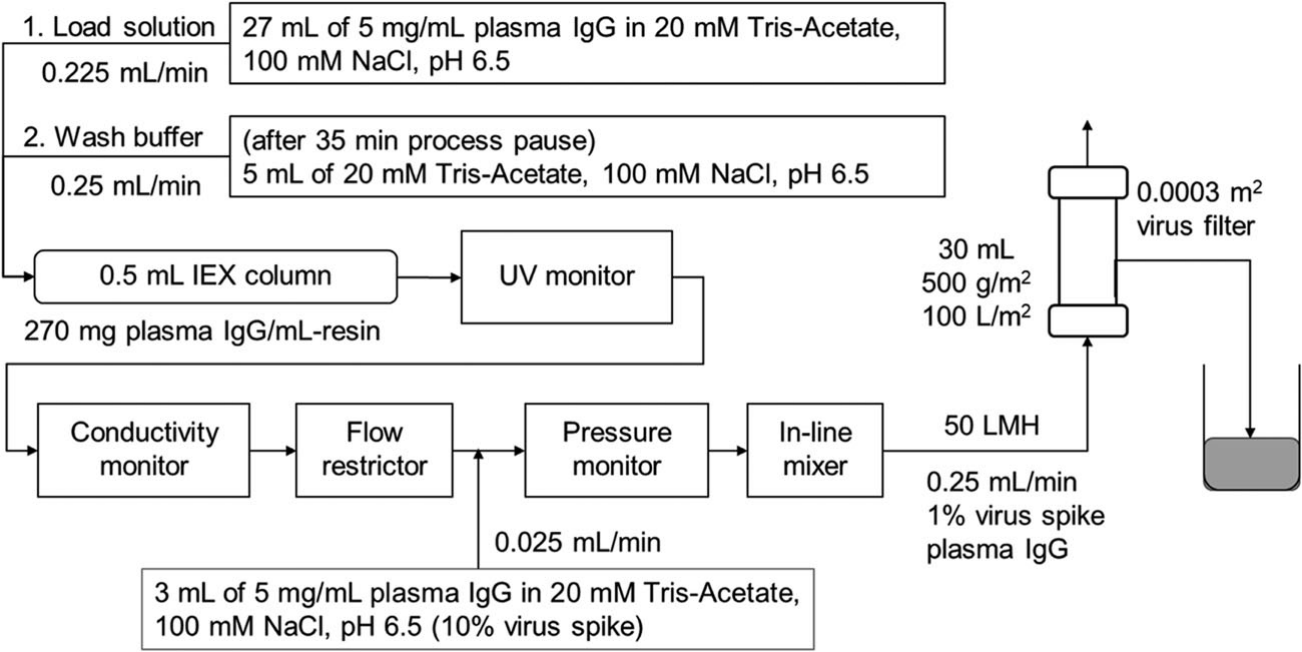
Setup for viral clearance test using an integrated process with in‐line spiking with virus (MVM or X‐MuLV) consisting of one IEX chromatography column (mixed‐mode AEX, grafted CEX, or modified CEX) and a virus filter connected in series for processing of plasma IgG solution (5 mg/ml plasma IgG in 20 mM Tris‐Acetate, 100 mM NaCl, pH 6.5). AKTA pure 25 was used for this setup

The in‐line spiking viral clearance study was designed and conducted by ViruSure using the setup and test conditions for loading protein solution from the chromatography column to the virus filter as shown in Figure 2. The two viruses used in this study, MVM and X‐MuLV, have different sizes. Virus spike solution prepared at 10% in plasma IgG solution with a concentration of 5 mg/ml adjusted to 20 mM Tris‐Acetate, 100 mM NaCl, pH 6.5 buffer were passed through 0.1 μm PES prefilter for MVM and 0.2 μm PES prefilters for X‐MuLV before use. Each chromatography resin (mixed‐mode AEX, grafted CEX, or modified CEX) was packed in Tricorn 5/20 column (CV, 0.5 ml; 5 mm ID; GE Healthcare) and directly connected to a 0.0003 m^2^ Planova BioEX filter without pooling on an AKTA pure 25. The chromatography column and virus filter were independently washed and equilibrated before use. First, 27 ml of nonvirus spiked plasma IgG solution with a concentration of 5 mg/ml adjusted to 20 mM Tris‐Acetate, 100 mM NaCl, pH 6.5 buffer was supplied by system pump at a flow rate of 0.225 ml/min while 3 ml of 10% virus (MVM or X‐MuLV) spiked in the same protein solution was supplied by sample pump at a flow rate of 0.025 ml/min to the feed stream after passing through the chromatography column, UV monitor, conductivity monitor, and flow restrictor and before the in‐line mixer and filter. In these runs, 30 ml of 1% virus spiked protein solution was fed to the virus filter at a constant flow rate of 0.25 ml/min, producing a flux of 50 LMH. The 1% virus spiked protein solution was confirmed by assay conducted after the runs to have log titers of 6.9 and 4.9 TCID_50_/ml for MVM and X‐MuLV, respectively. After reaching 30 ml of protein solution on the virus filter, both pumps were stopped for a 35 min process pause. Then, only the system pump was turned on and 5 ml of equilibration buffer was fed to the setup at a flow rate of 0.25 ml/min to wash out the residual protein solution from the chromatography column and the virus filter. Using this procedure, a viral clearance test at a constant flow rate was conducted, and the effect of a process pause was evaluated. The load was 270 mg plasma IgG/ml‐resin or 54 ml/ml‐resin for each column and 500 g plasma IgG/m^2^ or 100 L/m^2^ for the virus filter.

### 2.10 Viral clearance for a virus filter at low flux with plasma IgG

**Figure 3:**
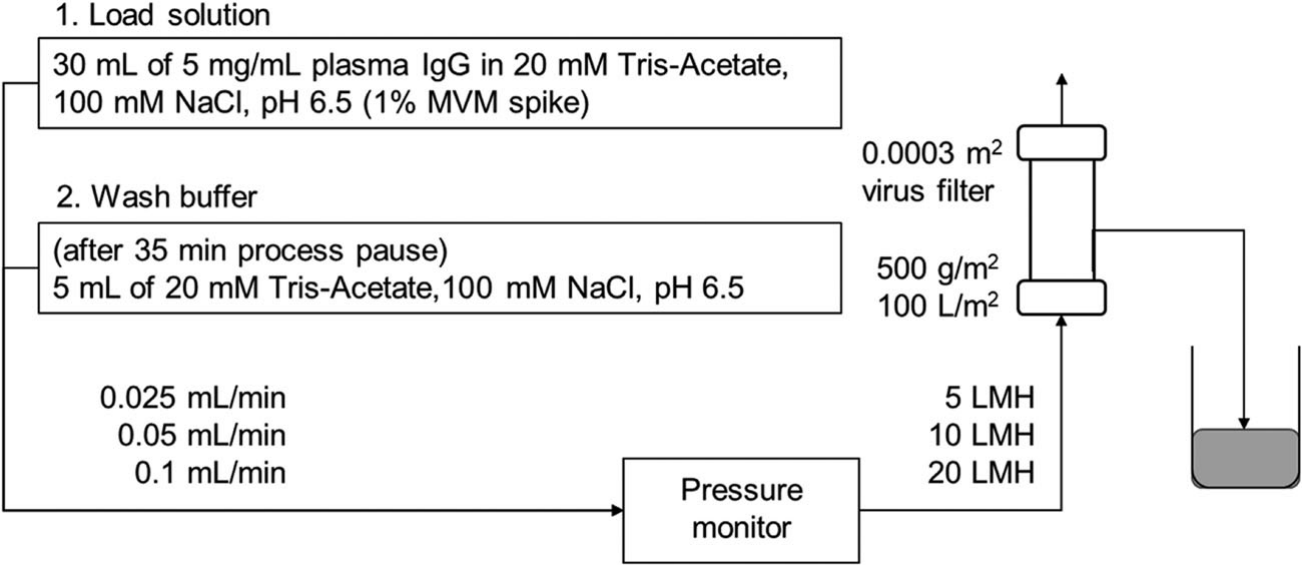
Setup for the constant flow rate MVM clearance test with plasma IgG (5 mg/ml plasma IgG in 20 mM Tris‐Acetate, 100 mM NaCl, pH 6.5). Filtration runs were conducted at 5, 10, and 20 LMH

The low flux viral clearance study of Planova BioEX filter was conducted by ViruSure. The setup and test conditions are shown in Figure 3. In this study, 30 ml (100 L/m^2^) of 5 mg/ml plasma IgG in 20 mM Tris‐Acetate, 100 mM NaCl, pH 6.5 solution spiked with 1% MVM (0.1 μm prefiltered) was filtered at 5, 10, and 20 LMH. The log titers of MVM were 7.23, 7.38, and 7.56 TCID_50_/ml for the 5, 10, and 20 LMH runs, respectively. Following plasma IgG solution filtration and a 35 min process pause, 5 ml of equilibration buffer wash was conducted. Viral clearance was measured for the plasma IgG solution permeate and buffer wash permeate.

### 2.11 Blocking model analysis

Filtration behavior of the virus filter at a constant flow rate was evaluated with the following four blocking models: cake filtration, intermediate blocking, standard blocking, and complete blocking. Blocking models assume the filter to be a collection of cylindrical pores with uniform length and inner diameter. While no filter has this simplified pore structure, we can expect to see differences in filtration behavior for the different blocking models that are representative of the processes. Four blocking models are described as below (Grace, 1956; Sumiya, 2013).

### 2.12 Cake filtration model

In this blocking model, the substances causing clogging do not block the cylindrical pores but rather adhere to the surface of the filter, causing the formation of flow paths that are new and different from those of the filter material.

### 2.13 Intermediate blocking model

In this blocking model, the substances causing clogging accumulate on already trapped substances and the entrances of the cylindrical pores. The substances causing clogging are distributed between the entrance of the pores and already clogged pores.

### 2.14 Standard blocking model

In this blocking model, the substances causing clogging are distributed evenly over the inner surface of the cylindrical pores, and the inner diameter of the pores gradually becomes smaller.

### 2.15 Complete blocking model

In this blocking model, the substances causing clogging accumulate at the entrance of the pores, and accumulation continues until all cylindrical pores become completely clogged.

In these models, filtration behavior can be expressed theoretically by calculating the change in flow resistance based on the types of substances causing clogging. Equations expressing the relationship between filtration volume and pressure for constant flow rate filtrations for the four blocking models, which differ by the condition of the retained substances that cause clogging, are shown in Table 1 (Grace, 1956; Sumiya, 2013), where *p*_0_ is the initial transmembrane pressure, *p* is the transmembrane pressure at the filtration volume, *V*, and *k* is the plugging constant specific to the filter and solution being filtered.

### 2.16 Blocking model fit analysis

Filtration volume, *V*, and pressure obtained from the filtration experiments were applied to Equations (2), (4), (6), and (8). The plugging constant, *k*, was determined by calculating the slope of the line obtained by applying the least‐squares method to obtain the line of best fit of the plot of the left side of each equation against *V*. Equation (9) was used to calculate the average pressure difference (*Δp*) between the experimental value of the filtration pressure obtained from filtration experiments and the filtration pressure obtained from each blocking model.

(9) $$\mathrm{average}∆p=\frac{1}{N}\sum_{i=1}^{N}\sqrt{{\left(p_{\exp,i}-p_{\mathrm{cal},i}\right)}^{2}}$$

where *p*_*exp,i*_ and *p*_*cal,i*_ are the *i*th filtration behavior measurement for experimental and calculated pressure values and *N* is the number of experimental measurements for each solution.

## 3 RESULTS AND DISCUSSION

### 3.1 Integration of flow‐through chromatography and filtration in a mab process

**Figure 4:**
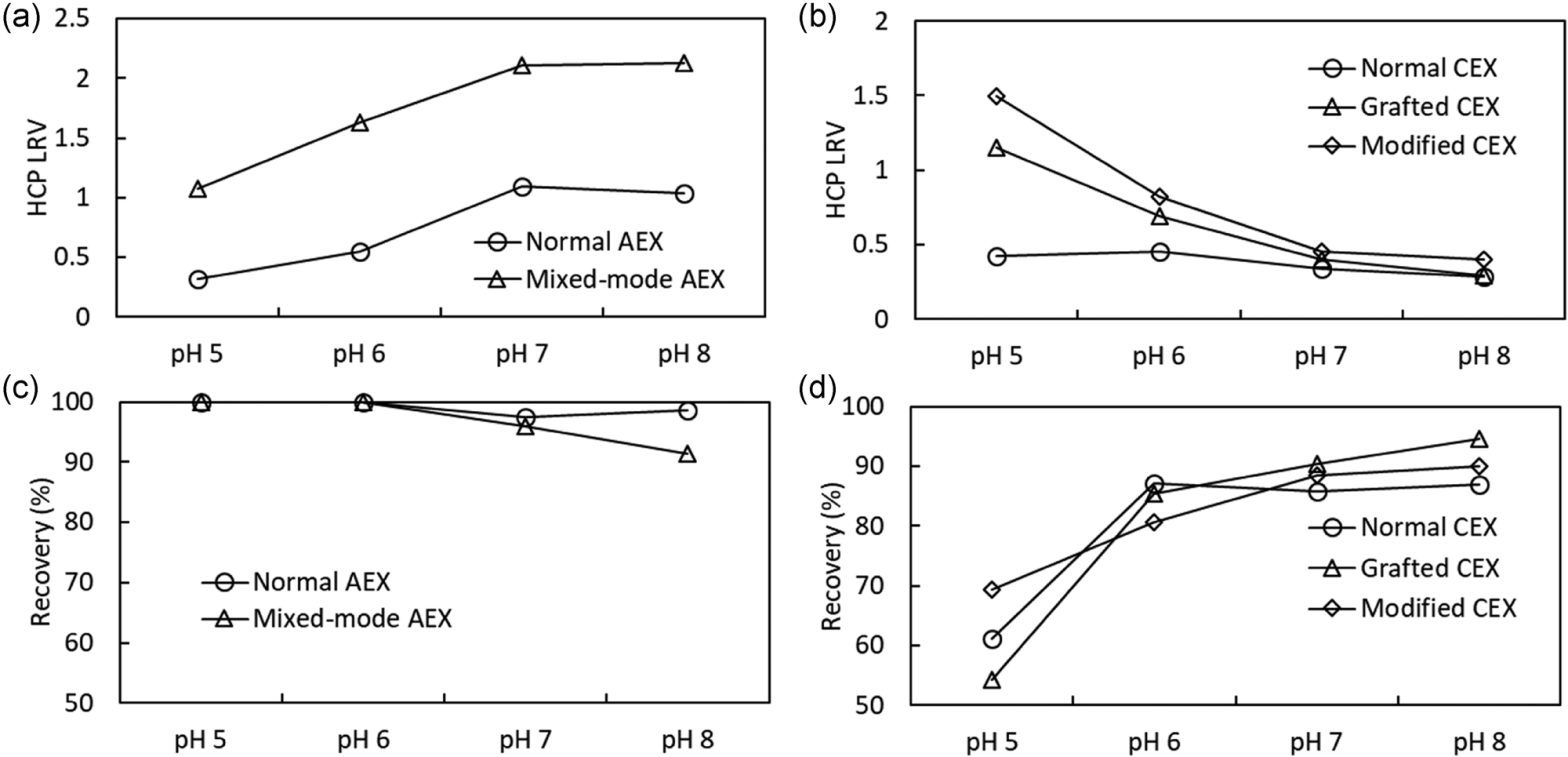
HCP reduction and protein recovery for flow‐through processing of mAb solution with 0.5 ml CV AEX and CEX column chromatography. (a) HCP reduction with AEX. (b) HCP reduction with CEX. (c) protein recovery with AEX. (d) protein recovery with CEX. For all runs, mAb at 10 mg/ml in 20 mM Tris‐Acetate, 100 mM NaCl, pH 5, 6, 7, or 8 was processed at 0.25 ml/min (0.5 CV/min) with 200 mg/ml‐resin load. HCP concentration in the load solution was about 3000 ng/mL for AEX column chromatography runs and 1500 ng/ml for CEX column chromatography runs. HCP, host cell protein; mAb, monoclonal antibodies

Chromatography processing with AEX (normal AEX and mixed‐mode AEX) and CEX (normal CEX, grafted CEX, and modified CEX) resins used separately at various pH values was conducted in a preliminary study to determine the resins and optimal buffer conditions to be used in the integrated process. For all runs, 10 mg/ml mAb was used with a starting HCP concentration of about 3000 ng/ml for AEX column chromatography runs, and about 1500 ng/ml for CEX column chromatography runs. The results in Figure 4 show a trade‐off between HCP removal and protein recovery as pH is adjusted. For AEX resins, HCP LRV on mixed‐mode AEX was around 1 at pH 5 and around 2 at pH 7, which is markedly higher than for normal AEX, which had a maximum HCP LRV of around 1 at pH 7 and 8. These results are comparable with published data (Kang et al., 2015), in which HCP LRV by AEX flow‐through processing is around 1 at similar conductivity and pH conditions. For CEX resins, modified CEX showed the highest HCP removal rate, followed by grafted CEX and normal CEX, and these differences were more pronounced at lower pH. For modified CEX, HCP LRV was 1.5 at pH 5 but decreased to around 0.5 at pH 7. While AEX resins showed high protein recovery (≥95% at pH 5–7), protein recovery for CEX was around 70% or less at pH 5 and reached 80%–90% at pH 6–8.

**Figure 5:**
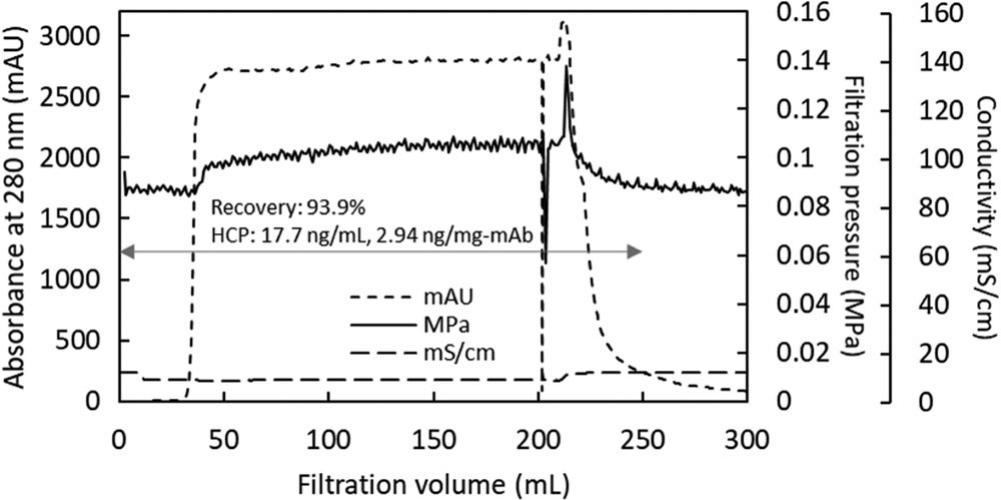
Absorbance of UV at 280 nm, filtration pressure, and conductivity profiles obtained from the mAb processing setup shown in Figure 1. Protein recovery and HCP concentration are noted in the figure. mAb at 10 mg/ml in 20 mM Tris‐Acetate, 9 mS/cm, pH 6.5 with 3800 ng/ml HCP and a buffer wash were processed. HCP, host cell protein; mAb, monoclonal antibodies

Considering the trade‐off between HCP removal and protein recovery for these two resin types along with the dependence on pH, mixed‐mode AEX and modified CEX were selected for use in series with 20 mM Tris‐Acetate, 100 mM NaCl, pH 6.5 buffer at constant flow rate on the setup shown in Figure 1. The profiles of UV absorbance, filtration pressure, and conductivity during the process (Figure 5) show an increase in pressure when the protein solution reaches the virus filter after displacing the equilibration buffer from the system piping and both chromatography columns. Thereafter, UV absorbance and filtration pressure remained stable, and after switching back to equilibration buffer following the predetermined load of protein, there was a momentary pressure drop at 200 ml. As protein was pushed out from the column by the equilibration buffer wash (50 ml), the protein concentration decreased and the filtration pressure also gradually decreased. For the total collected volume of 250 ml, good protein recovery of 93.9% and good HCP removal to 17.7 ng/ml (2.94 ng/mg‐mAb or 2.94 ppm, HCP LRV of 2.3) were achieved. Comparing this HCP removal to literature values, bind and elute CEX and flow‐through AEX membrane processing operated in continuous modes (David et al., 2020) achieved HCP reduction from about 100 ppm to about 10 ppm with HCP LRV around 1. The load capacity on the CEX column was 22 mg/ml‐resin and that of the AEX membrane was less than 470 mg/ml‐membrane with a flow rate of 0.5 membrane‐volume/min. In another study using a fully connected flow‐through AEX‐CEX process (Ichihara et al., 2019), HCP was reduced from 567 ppm to about 30 ppm and HCP LRV around 1.3 was achieved. The load capacity on the CEX column was 984 mg/ml‐resin and that of the AEX column was 492 mg/ml‐resin with a flow rate of 12 CV/h for AEX. Though the load capacity and flow rate in these two references are different from the 380 mg/ml‐resin and 2.4 CV/h used in our study, these results indicate that comparable HCP removal was obtained in this study.

The pressure profile of the virus filter during mAb loading shown in Figure 5 is stable and well below the maximum operating pressure of Planova BioEX of 0.343 MPa, suggesting that load capacity much higher than 540 L/m^2^ may be expected. These results demonstrate that columns in‐series can be effectively integrated with virus filtration. Constant flow rate protein solution loading with an integrated process consisting of column chromatography and virus filtration connected directly without pooling is an efficient process in which the columns effectively remove impurities, allowing stable filtration pressure for the integrated virus filtration.

In this study, we conducted both AEX and CEX in flow‐through mode to simplify the setup for this proof of concept. However, because CEX is often conducted in bind and elute mode, future studies will be needed to evaluate the feasibility of integrated processes with flow‐through AEX and bind and elute CEX connected to virus filtration.

### 3.2 Characterization of pressure in filtration of plasma IgG

**Figure 6:**
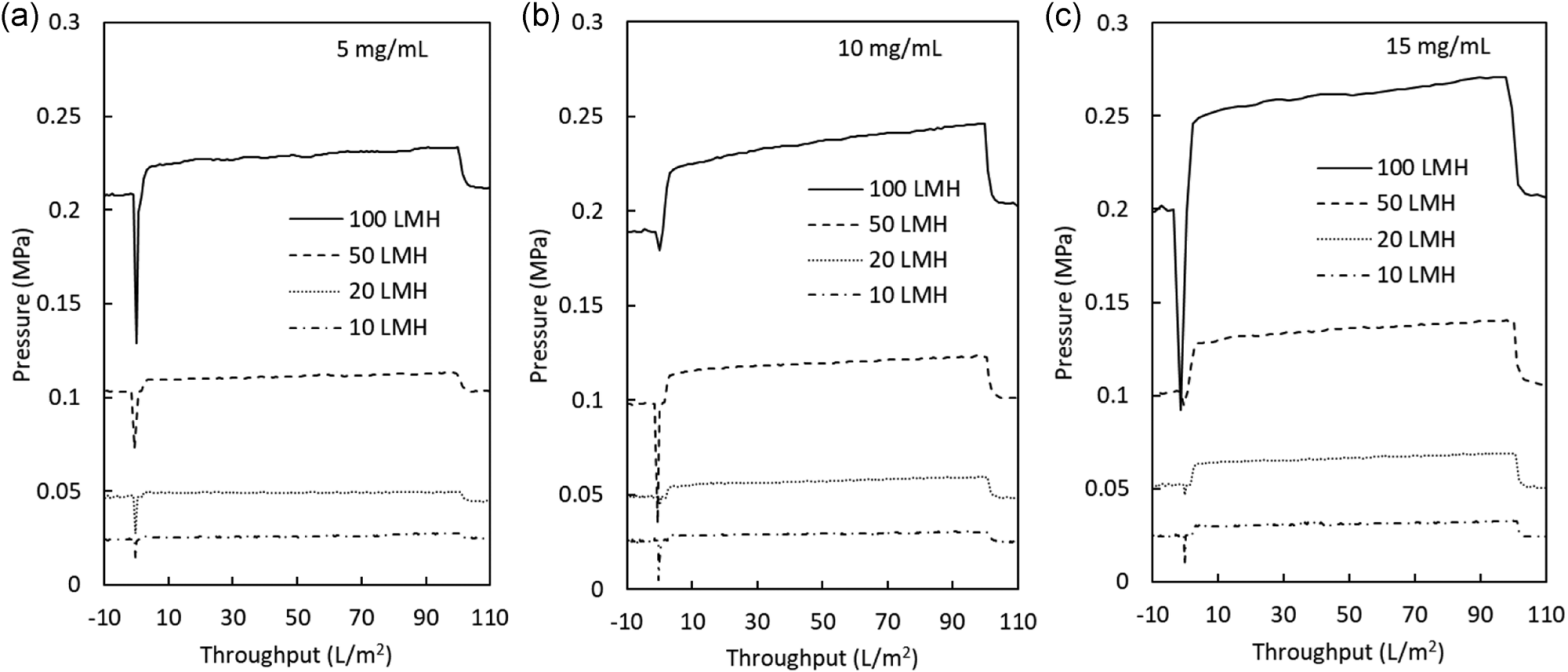
Transmembrane pressure during filtration of plasma IgG solution shown as (a) 5 mg/ml plasma IgG, (b) 10 mg/ml plasma IgG and (c) 15 mg/ml plasma IgG. For all filtrations, 100 L/m^2^ of plasma IgG at 5, 10, or 15 mg/ml in 20 mM sodium acetate, 100 mM NaCl, pH 5.0 was filtered at 10, 20, 50, or 100 LMH

The filtration behavior (pressure) for 5, 10, and 15 mg/ml plasma IgG solution on a virus filter under constant flow rate corresponding to 10, 20, 50, and 100 LMH is shown in Figure 6. Although the throughput of 100 L/m^2^ is lower than most practical manufacturing processes, all runs maintained pressure that was well below the maximum operating pressure of 0.343 MPa for the Planova BioEX filter. Thus, the filter is capable of much higher throughput, especially when filtration can be conducted at low flux. In another recent study, a throughput of 5200–6900 L/m^2^ was achieved for filtration of human immunoglobulin (h‐IgG) on a Planova BioEX filter at a constant flow rate (72 LMH) (Lute et al., 2020) albeit with a low protein concentration of 0.025 mg/ml. In Figure 6, following a pressure dip at 0 L/m^2^ due to switching from equilibration buffer (20 mM sodium acetate, 100 mM NaCl, pH 5.0) to the protein solution, the filtration pressure was stable throughout the protein filtration, followed by a decrease in pressure at 100 L/m^2^ when the feed solution was changed to the equilibration buffer. The slight pressure increases were mostly proportional and were higher with higher flux and protein concentration. For filtrations conducted at 20 LMH, the filtration pressure was extremely low and almost no pressure increase during the run was observed. For all plasma IgG concentrations and flow rates, the filtration pressure was stable, and there were no irregular pressure changes during protein filtration in this test, demonstrating the robustness of the virus filter used for this test.

### 3.3 Viral clearance for integrated systems with plasma IgG

The in‐line spiking methods that have been demonstrated as being suitable for constant pressure filtrations (Genest et al., 2013; Wu et al., 2008) may also be applicable to integrated processes with column chromatography. To conduct viral clearance tests for continuous constant flow rate processes, some adjustments from a constant pressure setup are needed. Following the strategy for constant pressure processing with in‐line spiking, a pump was used to supply a concentrated virus spike (X‐MuLV or MVM) in plasma IgG to the feed stream after the chromatography column (mixed‐mode AEX, grafted CEX, and modified CEX) and before the filter without pooling (Figure 2). X‐MuLV and MVM removal by the Planova BioEX filter following processing in an integrated column chromatography process shows complete removal (no virus was detected in the TCID_50_ assay) for all three resins with virus LRV greater than 3.12 for X‐MuLV and greater than 5.13 for MVM for both the flow‐through fraction and flow‐through with wash (Table 2). The transmembrane pressures (TMP) during the loading was about 0.13 MPa and pressure increase was within 0.01 MPa for the load capacity of 100 L/m^2^ for all tests. These stable pressure profiles suggest high stability of constant flow rate loading and robust filterability of the Planova BioEX filter. It is also suggested that our study conditions afford a larger maximum load capacity than previous reports that used constant pressure loading and a different virus filter, which showed significant flux decay at 100 L/m^2^ throughput (Ireland et al., 2005; Khan et al., 2009). It is noted that the X‐MuLV LRV was low due to a low load titer of X‐MuLV (4.9 log TCID_50_/ml), but all runs showed complete viral clearance. This is supported by the report that X‐MuLV LRV was lower than that of MVM LRV for viral clearance tests because of the lower starting titer for X‐MuLV (Gefroh et al., 2014). These results demonstrate that under the test conditions in this study, the virus removal capability of Planova BioEX filter connected to the chromatography column is robust. Additionally, no virus was detected in the permeate collected after the 35 min process pause, confirming that the virus filter used in the test has a very robust virus removal capability despite having a process pause.

**Table 2:** X‐MuLV and MVM clearance for constant flow rate in‐line spiking test of 5 mg/ml plasma IgG in 20 mM Tris‐acetate, 100 mM NaCl, pH 6.5 for integrated processes consisting of chromatography and virus filtration

		Virus LRV† (log TCID_50_/ml)		
Virus spike		Mixed‐mode AEX⇨Planova BioEX	Grafted CEX⇨Planova BioEX	Modified CEX⇨Planova BioEX
X‐MuLV	Flow‐through fraction	≥3.75	≥3.62	≥3.50
	Flow‐through + Wash	≥3.39	≥3.26	≥3.12
MVM	Flow‐through fraction	≥5.56	≥5.50	≥5.56
	Flow‐through + Wash	≥5.19	≥5.13	≥5.19

^†^ Log titers of X‐MuLV and MVM loaded onto the virus filter were 4.9 and 6.9 TCID_50_/ml, respectively.

To confirm the virus removal capability of the chromatography column, a viral clearance test was conducted with the same load to each of the mixed‐mode AEX, grafted CEX and modified CEX chromatography columns with a flow rate of 0.25 ml/min (0.5 CV/min or 30 CV/h). For these three resins, the X‐MuLV LRV of the flow‐through fraction excluding the equilibration buffer fraction was 2.25, 0.13, and 0.44 for a load capacity of 150 mg/L‐resin and 1.96, 0.21, and 0.38 for a load capacity of 300 mg/ml‐resin, respectively. For MVM, LRV of the flow‐through fraction was 3.94, −0.25, and 0.44 for a load capacity of 150 mg/L‐resin and 2.99, −0.16, and 0.45 for a load capacity of 300 mg/ml‐resin, respectively. All runs were conducted at pH 6.5, which is above the isoelectric point (pI) of 5.8 for X‐MuLV and 6.2 for MVM, as reported by Strauss et al. (2009). Because the solution pH was higher than the virus pI, low or no virus removal for the CEX flow‐through mode runs are expected and this low CEX virus reduction might be a concern in this setup. AEX runs resulted in higher virus LRV for the lower load capacity (150 mg/L‐resin) runs for both tested viruses. In literature, for AEX resins run using flow‐through mode with mAb load capacity of 50 mg/ml‐resin, X‐MuLV LRV was 2.4–3.0 and PPV LRV (PPV belongs to parvovirus like MVM) was 2.1–5.9 as reported by Miesegaes et al. (2014). The virus LRV for the AEX resin in this study, in which load capacity was higher than 50 mg/ml‐resin (150 and 300 mg/ml‐resin), are roughly comparable with the reported values. For improving the virus reduction in the total flow‐through column process proposed in this study, decreasing load capacity, loading at a lower flow rate, or selection of pH to optimize virus removal may be effective.

Operating conditions are generally adjusted to optimize chromatography processes. In this study, the chromatography conditions chosen were intended to allow for simple chromatography operation, albeit in less than ideal conditions. Operating at a pH greater than 7 would give a greater HCP removal for mixed‐mode AEX and may also improve virus reduction, but this adjustment would result in lower HCP LRV for modified CEX and a slight decrease in protein recovery. Other possibilities for achieving higher virus reduction include using a smaller load amount (mg/ml‐resin) and running at a lower flow rate. However, the former may decrease recovery and the latter may result in a longer process time. These trade‐off relationships require careful consideration when designing a commercial continuous manufacturing process. In this study, the conditions showcased the robustness of virus filtration as a necessary component of these processes, ensuring viral safety under a wide range of solution conditions (Lute et al., 2020; Strauss et al., 2017).

### 3.4 Viral clearance at low flux with plasma IgG

Considering the possibility of a continuous process that connects perfusion cell culture, multicolumn chromatography systems, and continuous low pH virus inactivation over long durations, virus filtration conducted over a long duration at low flow rates with stable pressure is required.

To evaluate the effects of constant and low flow rate filtration with a process pause on virus removal, we used plasma IgG spiked with MVM at flow rates corresponding to 5, 10, and 20 LMH and a 35 min process pause followed by a wash with 5 ml of equilibration buffer as shown in Figure 3. MVM was used for the test because it has a small size, and there is a concern for virus breakthrough with low flow rate filtrations. The volume of protein solution fed to the filter was 30 ml for all runs, and filtration duration was 1200, 600, and 300 min, respectively. The TMP during protein loading were 0.02–0.024 MPa for 5 LMH, 0.04–0.055 MPa for 10 LMH, and 0.05–0.065 MPa for 20 LMH for load capacity of 100 L/m^2^, respectively. These stable TMP values suggest that the virus spike solution included no particular impurities that clogged the filter. All runs, including the wash after the process pause, showed good viral clearance with MVM LRV of higher than 5, and no virus was detected from the filtrate of any of the runs (Table 3). Planova BioEX filters used in this study showed robust virus removal capabilities even with a process pause and the low flux with low pressure and long duration conditions expected in continuous processes.

**Table 3:** MVM clearance of 30 ml of 5 mg/ml plasma IgG in 20 mM Tris‐acetate, 100 mM NaCl, pH 6.5 for virus filtration (0.0003 m^2^) at various constant flow rates

	MVM LRV† (log TCID_50_/ml) at various flow rates		
Flow rate	0.025 ml/min	0.05 ml/min	0.1 ml/min
	(5 LMH)	(10 LMH)	(20 LMH)
Filtrate sample	≥5.27	≥5.40	≥5.59
Filtrate sample + Wash	≥5.13	≥5.24	≥5.43

^†^ The log titers of MVM loaded onto the virus filter were 7.23, 7.38, and 7.56 TCID_50_/ml for the 5, 10, and 20 LMH runs, respectively.

### 3.5 Blocking model analysis of integrated process with plasma IgG and mAb

**Figure 7:**
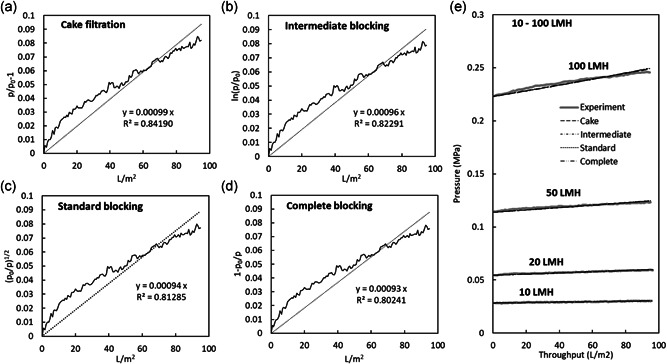
Plugging constant determination using the least‐squares method (a)–(d) and plots of experimental values and calculated values using each blocking model (e) for filtration of plasma IgG solution with a virus filter: (a) cake filtration model using Equation (2) at 50 LMH, (b) intermediate blocking model using Equation (4) at 50 LMH, (c) standard blocking model using Equation (6) at 50 LMH, (d) complete blocking model using Equation (8) at 50 LMH, and (e) pressure profiles for experimental values and calculated values using each blocking model at 10, 20, 50, and 100 LMH. For all filtrations, plasma IgG at 10 mg/ml in 20 mM sodium acetate, 100 mM NaCl, pH 5.0 was filtered

Evaluation of filtration behavior with blocking models allows characterization of the process for further process development. In these plasma IgG and mAb processes controlled with constant flow rate, plotting the experimental and calculated filtration pressure against the throughput gives insights into the clogging mechanism and the potential capacity for processing at larger scales. Using the 10 mg/ml plasma IgG filtrations shown in Figure 6b, plots for determining plugging constant *k* for 50 LMH applied to each blocking model (cake, intermediate, standard, and complete) are shown in Figure 7a–d, and the experimental pressure increase over the run along with profiles calculated for each model are shown for 10, 20, 50, and 100 LMH runs in Figure 7e. Figure 7e shows that the calculated filtration pressure profiles for all blocking models were nearly identical up to 100 L/m^2^ throughput, which is a reflection of very little filter clogging under the filtration conditions used in this study. As the filtration pressure is markedly lower than the suggested maximum operating pressure of 0.343 MPa for the Planova BioEX filter, pressure data from even larger throughputs will be needed to evaluate the predictive ability of these blocking models for larger constant flow rate processes.

**Figure 8:**
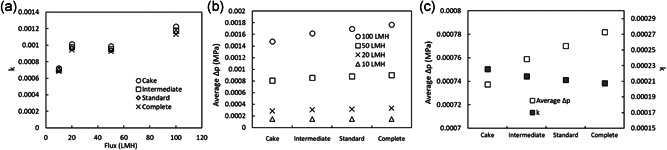
Blocking model analysis for filtration of plasma IgG (a) and (b) and mAb solution (c) with a virus filter. (a) Plugging constant, *k*, of each blocking model noted in the panel and (b) average pressure difference between experimental values and calculated values, *Δp*, for 10 mg/ml plasma IgG solution filtration with 10, 20, 50, and 100 LMH shown in Figure 6b. (c) Plugging constant, *k* and *Δp* of each blocking model for 10 mg/ml mAb solution processing with 40 LMH and a throughput of 540 L/m^2^ on the integrated process shown in Figure 5. *Δp* is calculated using Equation (9). Blocking models: cake filtration, intermediate blocking, standard blocking, and complete blocking

Plugging constant was shown to be proportional to the volume of the substances retained by the filter (Grace, 1956; Sumiya, 2013), and a larger variety of substances are expected to be retained by a filter operated with higher flux. The relationship with flux for plugging constants obtained for 10 mg/ml runs shows a roughly proportionally increasing trend with higher flux (Figure 8a). The calculated pressure profiles did not differ with the blocking model, likely due to the very minimal clogging that occurred in these runs. Plotting the difference in filtration pressure between experimental values and modeling results for the 10–100 LMH runs calculated using Equation (9) reveals the slight differences among the blocking models (Figure 8b), showing that the cake filtration model has the closest fit with the experimental values and that the largest differences between models are at higher fluxes, though the differences were small.

For the integrated mixed‐mode AEX and modified CEX mAb process with a Planova BioEX filter, blocking model analysis was applied to the 540 L/m^2^ portion of the mAb filtration (up to 200 ml with 40 LMH shown in Figure 5). As shown in Figure 8c, the average difference in filtration pressure between experimental values and modeling results was the smallest for the cake filtration model for mAb. Although differences between models were small, the best fit to the cake filtration model may be attributed to the stable pressure profile and small increase of TMP without significant clogging. Even for this much larger throughput of 540 L/m^2^ for the mAb process compared to 100 L/m^2^ for the plasma IgG process, the plugging constant, *k*, for each of the blocking models for the mAb was almost one order of magnitude smaller than for plasma IgG (k is about 0.001 for 50 LMH as shown in Figure 8a), and all were similar with 0.000225 for cake filtration, 0.000216 for intermediate blocking, and 0.000212 for standard blocking and 0.000207 for complete blocking models. Thus, the mAb solution processed by mixed‐mode AEX and modified CEX column chromatography had a higher filterability than the commercial‐grade purified plasma IgG applied directly to the virus filter used in this study. Although no impurity data is available for the plasma IgG used in this study, future studies may benefit from measuring HCP and DNA impurities in plasma IgG that may cause clogging in the virus filter, which may explain the higher k for plasma IgG solution. Another possible explanation may be that the lower filterability for plasma IgG is attributed to plasma IgG aggregates, as we have in‐house data showing that even a small plasma IgG aggregate spike of 0.05% results in significantly reduced filterability (unpublished data).

According to Figure 8c, the cake filtration model is the closest fit with experimental results, suggesting that a high throughput can be expected for the Planova BioEX virus filter used in an integrated process.

It is also worth considering whether combined blocking models suggested for constant pressure filtration processes (Bolton & Apostolidis, 2017; Bolton et al., 2006; Ho & Zydney, 2000) are also applicable to constant flow rate conditions.

## 4 CONCLUSIONS

We confirmed that integrating a chromatography step without pooling before the virus filtration step can be used to effectively improve the filterability of the protein solution in a virus filtration operated at low flux in constant flow rate mode. In a mAb process the combination of a mixed‐mode AEX and modified CEX column used in‐series effectively removed HCP with high protein recovery. For the viral clearance test using plasma IgG, even for long duration filtration at low flux with a process pause, a high virus removal capability was confirmed. Our findings suggest the usefulness of blocking model analysis to characterize virus filtration properties and demonstrate the robust and high filterability of the virus filter used in this study. Thus, the Planova BioEX filter is highly applicable for continuous processing. This integration could lead to a large reduction in footprint and process time in DSP processes and is significant for realizing an efficient continuous process.

## AUTHOR CONTRIBUTIONS

**Hironobu Shirataki**: Conceptualization, Formal analysis, Visualization, Writing ‐ Original Draft, Writing ‐ Review & Editing. **Yoshiro Yokoyama**: Methodology, Investigation. **Hiroki Taniguchi**: Methodology, Investigation. **Miku Azeyanagi**: Investigation.

## Data Availability

The data that support the findings of this study are available from the corresponding author (HS), upon reasonable request.
